# Targeting the Microbiota to Address Diet-Induced Obesity: A Time Dependent Challenge

**DOI:** 10.1371/journal.pone.0065790

**Published:** 2013-06-07

**Authors:** Siobhan F. Clarke, Eileen F. Murphy, Orla O’Sullivan, R. Paul Ross, Paul W. O’Toole, Fergus Shanahan, Paul D. Cotter

**Affiliations:** 1 Alimentary Pharmabiotic Centre, University College Cork, Cork, Ireland; 2 Teagasc Food Research Centre, Moorepark, Fermoy, Cork, Ireland; 3 Microbiology Department, University College Cork, Cork, Ireland; 4 Alimentary Health Ltd., Cork, Ireland; 5 Department of Medicine, University College Cork, Cork, Ireland; Charité-University Medicine Berlin, Germany

## Abstract

Links between the gut microbiota and host metabolism have provided new perspectives on obesity. We previously showed that the link between the microbiota and fat deposition is age- and time-dependent subject to microbial adaptation to diet over time. We also demonstrated reduced weight gain in diet-induced obese (DIO) mice through manipulation of the gut microbiota with vancomycin or with the bacteriocin-producing probiotic *Lactobacillus salivarius* UCC118 (Bac^+^), with metabolic improvement achieved in DIO mice in receipt of vancomycin. However, two phases of weight gain were observed with effects most marked early in the intervention phase. Here, we compare the gut microbial populations at the early relative to the late stages of intervention using a high throughput sequencing-based analysis to understand the temporal relationship between the gut microbiota and obesity. This reveals several differences in microbiota composition over the intervening period. Vancomycin dramatically altered the gut microbiota composition, relative to controls, at the early stages of intervention after which time some recovery was evident. It was also revealed that Bac^+^ treatment initially resulted in the presence of significantly higher proportions of *Peptococcaceae* and significantly lower proportions of *Rikenellaceae* and *Porphyromonadaceae* relative to the gut microbiota of *L. salivarius* UCC118 bacteriocin negative (Bac^-^) administered controls. These differences were no longer evident at the later time. The results highlight the resilience of the gut microbiota and suggest that interventions may need to be monitored and continually adjusted to ensure sustained modification of the gut microbiota.

## Introduction

Obesity is due to a surplus of energy intake over expenditure, resulting in storage of excess energy as fat. However, this is only part of a bigger story; an emerging theme is the relationship between the composition and functionality of microorganisms in the gut with obesity. [1], [2], [3], [4], [5] A corollary to this is the potential for manipulation of the gut microbiota in the prevention and management of obesity and associated metabolic disorders.

We previously showed that compositional changes in the faecal microbiota associated with diet-induced obesity are time-dependent and unrelated to markers of energy harvest, which change over time. [6] Furthermore, we have previously investigated the impact of administering the glycopeptide antibiotic vancomycin and the bacteriocin-producing probiotic *Lactobacillus salivarius* UCC118 (Bac^+^) to diet-induced obese (DIO) mice. Vancomycin resulted in an improvement in the metabolic abnormalities associated with obesity, including a significant reduction in weight gain, by the end of the intervention period. In contrast, when compared with an isogenic non-bacteriocin producing control (Bac^-^), the *L. salivarius* UCC118 Bac^+^ strain alters the gut microbiota but did not significantly alter metabolic markers or weight gain as measured at the end of the intervention period. [7].

While our initial report focused on the metabolic changes evident upon completion of the intervention strategies, the temporal changes in the microbiota need to be addressed further. The impact of vancomycin intervention on weight gain was most considerable during the early stages of intervention and a significant reduction in weight gain in mice fed with the Bac^+^ strain was apparent when compared with their Bac^-^ fed counterparts. [7] Here, we analyse and compare the gut microbial populations of these animals at the early (week 2) with the late (week 8) intervention period. The results reflect the resilience of the gut microbiota and show that therapeutic manipulation of the microbiota is likely to be more complex than anticipated with sustained adjustment likely to require multiple interventions over time.

## Materials and Methods

### Animals

3–4 week old male C57BL/6j mice were acquired from Harlan (oxon, UK) and housed within the biological services unit, University College Cork. UCC Animal Ethics Committee approved all experiments and experimental procedures were conducted under licence from the Irish government.

### Experimental Design

A low fat (lean) or high fat (DIO) diet was fed to male C57BL/J6 mice (aged 7 weeks) for 12 weeks followed by an intervention period during which the high fat diet was supplemented with the glycopeptide antibiotic vancomycin, the bacteriocin producing (Bac^+^) *L. salivarius* UCC118, its bacteriocin negative derivative (Bac^–^) or was unsupplemented (9–10 mice/cohort) for a period of 8 weeks. For full experimental design see figure S1 and Murphy *et al.* [7].

### DNA extraction and High-throughput Amplicon Sequencing

Individual mouse faecal samples were collected and DNA was extracted on the same day of collection from fresh samples using the QIAmp DNA Stool Mini Kit (Qiagen, Crawley, West Sussex, UK) combined with an additional bead-beating step (30 s×3) and stored at −20°C. The microbiota composition of the samples was established by amplicon sequencing; universal 16 S rRNA primers estimated to bind to 94.6% of all 16 S genes (i.e. the forward primer F1 (5′-AYTGGGYDTAAAGNG) and a combination of four reverse primers R1 (5′-TACCRGGGTHTCTAATCC), R2 (TACCAGAGTATCTAATTC), R3 (5′-CTACDSRGGTMTCTAATC) and R4 (5′-TACNVGGGTATCTAATC) (RDP’S Pyrosequencing Pipeline: http://pyro.cme.msu.edu/pyro/help.jsp) were employed for PCR amplification. Molecular identifier tags were attached between the 454 adaptor sequence and the target-specific primer sequence, allowing for identification of individual sequences from the pooled amplicons. Ampure purification system (Beckman Coulter, Takeley, UK) was used to clean the amplicons before being sequenced on a 454 Genome Sequencer FLX platform (Roche Diagnostics Ltd, Burgess Hill, West Sussex, UK) in line with 454 protocols at the Teagasc high throughput sequencing centre. The amplicon sequences were deposited in the European bioinformatics institute sequence read archive (EBI-SRA) accession number ERP002448.

### Real Time Quantitative PCR

Total bacterial numbers (16 S rRNA gene copies per gram of wet stool) [8] were determined using real time quantitative PCR. The 16 S rRNA gene sequence of *E. coli* EPI300 was amplified using the universal 16 S primers 802R and 520F. [9] The amplified products purified using the High Pure PCR Cleanup Micro Kit (Roche Diagnostics Ltd, Burgess Hill, West Sussex, UK) were inserted into the pCR4-TOPO Vector (Invitrogen, Bio-Sciences, Dublin Ireland) and transformed into One Shot TOP10 Chemically Competent *E. coli* (Invitrogen, Bio-Sciences, Dublin, Ireland). Plasmids were extracted using the PureYield™ Plasmid Miniprep System (Promega, Madison, Wisconsin, USA) and quantified on the NanoDrop™ 1000 Spectrophotometer (Thermo Fisher Scientific, Waltham, Massachusetts, USA). Quantitative real time PCR (QPCR) was performed with SYBER-green (Roche Diagnostics Ltd, Burgess Hill, West Sussex, UK) on the lightcycler 480 (Roche Diagnostics Ltd, Burgess Hill, West Sussex, UK). The standard curve was generated using dilutions of the plasmid DNA. The following program was used to quantify total bacterial numbers: 95°C for 5 min followed by 40 cycles of 95°C for 20 s, 51°C for 20 s and 72°C for 20 s followed by melting curve analysis of 95°C for 5 s, 46°C for 1 min, and 97°C continuously and a final cooling at 40°C for 10 s. Samples contained 2 µl of PCR grade water, 1 µl of 520F (0.15 µM), 1 µl of the 802R (0.15 µM), 1 µl template DNA, and 5 µl of SYBR green. Samples and standards were run in triplicate. Negative controls were added to each plate with template DNA being replaced with PCR-grade water. The copy numbers of each sample were calculated from the standard curve and copies of 16 S rRNA/g wet stool was calculated using a previously outlined calculation. [8].

### Bioinformatics Sequence Analysis

A locally installed RDP pyrosequencing pipeline was used to quality trim the raw sequence data. Reads were removed that were shorter than the main distribution (150 bp for the 16 S rRNA V4 region), of low quality and not exact matches to barcoded tags and primer sequence. A locally installed version of SILVA 16 S rRNA database [10] was used to BLAST [11] the trimmed fasta sequence files using default parameters. Resulting BLAST output files were parsed through Megan [12] which uses a lowest common ancestor algorithm to assign reads to NCBI taxonomies. Prior to tree construction and summarization filtering was carried out within MEGAN using bit scores, similar to previous studies a bit-score cut-off of 86 was selected. [13], [14] Alpha diversity indices were generated using MOTHUR software. [15] Clustering of sequence reads into operational taxonomical units (OTUs) at 97% identity was achieved using QIIME suite software tools. [16] The ChimeraSlayer program was used to remove chimeric OTUs from aligned OTUs and the FastTreeMP tool generated a phylogenetic tree. [17], [18] Beta diversities were also calculated on the sequence reads based on weighted and unweighted unifrac and bray curtis distances; subsequently principal coordinate analysis (PCoA) and unweighted pair group method with arithmetic mean (UPGMA) clustering was performed on the samples. UPGMA clustering was visualised using Dendroscope software [19] while PCoA plots were viewed with KiNG viewer. [20] The nonparametric Kruskal-Wallis test [21] in the Minitab statistical package was employed to establish statistical significance (significance taken to be p≤0.05).

## Results

### α Diversity of the Murine Gut Microbiota Increases During the Intervention Period

**Figure 1:**
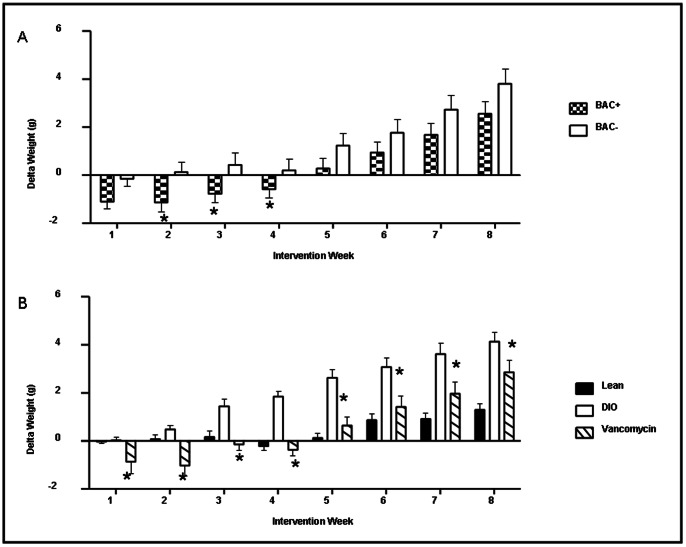
Delta weight gain over the eight week intervention period. (A) Bac^+^ intervention, when compared to Bac^-^ intervention, causes a significant reduction in weight gain in diet induced obese mice at weeks 2–4 (early intervention period) but this does not persist with time. (B) Vancomycin treatment results in a two phase reduction in weight gain in diet induced obese mice. In phase one (early; weeks 1–4) a significant reduction in weight gain relative to the initial start weight is observed. In the second phase, diet induced obese mice receiving vancomycin gain weight relative to the initial start weight but weight change continues to be significantly less than that in diet induced obese controls. Data represented as mean SEM n = 9–10 *p<0.05.

**Figure 2:**
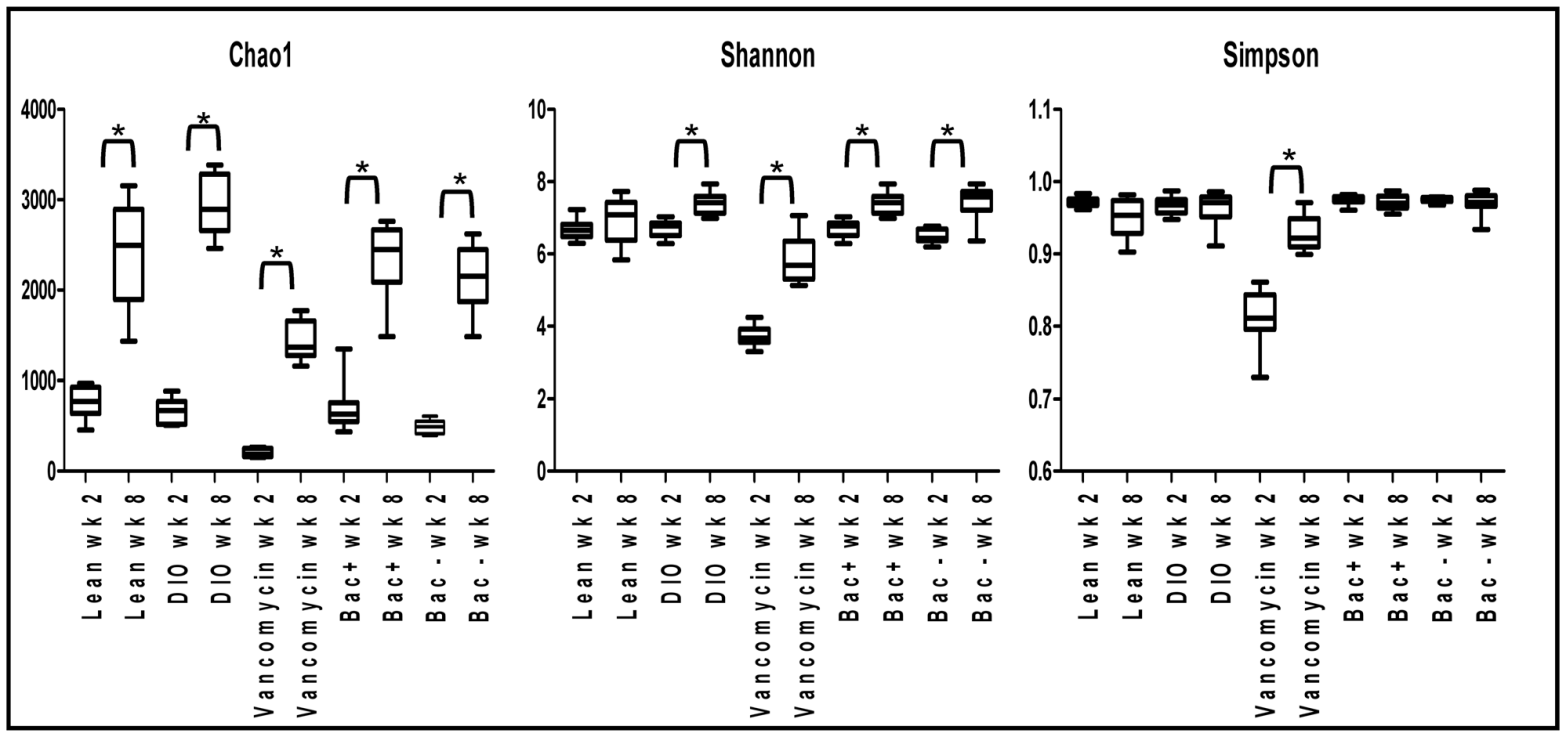
α diversity of the gut microbiota within each time point. In a number of instances significant increases in diversity are observed between intervention week 2 and week 8. Statistical significance was determined by kruskal wallis. * Statistical significant difference (p<0.05). Data represented as mean±SEM (n = 9–10).

There is a significant reduction in weight gain in DIO mice at intervention weeks 2–4 (early intervention period) in the Bac^+^ intervention, when compared to Bac^-^ intervention, but this does not persist with time (Figure 1A). [7] Vancomycin administration results in a two phase reduction in weight gain in DIO mice. In phase one (early intervention weeks 1–4) a significant reduction in weight gain relative to the initial start weight is observed. In the second phase, DIO mice receiving vancomycin gain weight relative to the initial start weight but weight change continues to be significantly less than that in DIO controls (Figure 1B). [7] The relationship between these early intervention period-specific observations and the gut microbiota were investigated through high throughput DNA sequencing. A total of 86,103 V4 16 S sequence reads, corresponding to faecal pellets from mice at intervention week 2 of the study, were generated. These corresponded to an average of 14,978 reads per group or 1,757 per mouse. These reads were analysed and compared with 212,655 16 S sequence reads generated from the mice at intervention week 8. [7] Shannon diversity, simpson diversity and species richness estimations were calculated for each data set (Figure 2). The Chao1 estimator of species richness reveals significant differences in species richness with time in all populations, including lean and DIO animals not exposed to interventions. Shannon diversity data revealed a high level of biodiversity in all groups along with a significant increase in diversity with time in all cohorts except those in receipt of a lean diet only (Figure 2). The Simpson diversity index-based analysis, which also takes account the number of species present and the relative abundance of each species, particularly highlighted the significant increase in diversity in animals in receipt of vancomycin as the intervention period continued. Thus while a general increase in gut microbial diversity was apparent as the mice aged, it was apparent that exposure to vancomycin brought about an initially considerable reduction in diversity which was diminished with time. Goods coverage ranged between 95% and 98% at week 2 and from 84% to 86% at week 8. Rarefaction curves were seen to be approaching parallel or parallel (Figure S2) signifying that extra sampling would yield a limited increase in species richness. Of the reads at week 2, 74,686 (87%) were assigned at phylum level, 59,720 (69%) at the family level and 40,947 (48%) at genus level.

### Taxonomical Analysis Highlights the Temporal Impact of Vancomycin on Specific Components of the Gut Microbiota

**Figure 3:**
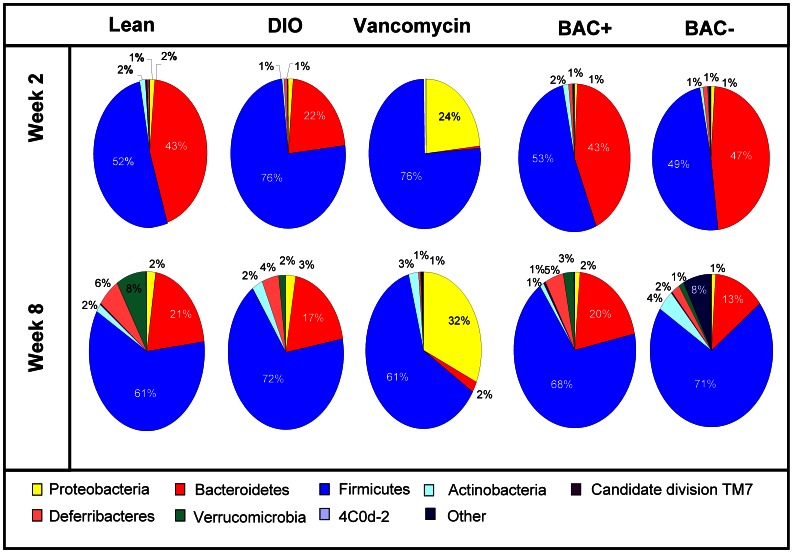
Microbial distribution at phylum level. Phylum level microbial distribution in all data sets at intervention week 2 and week 8. The pie charts represent total percentage read number for the corresponding colour coded phylum (*n* = 9–10 per group).

Analysis of the gut microbiota composition after 2 weeks established that the major difference, at the phylum level, between the microbiota of the lean and diet induced obese (DIO) mice is the presence of relatively greater proportions of Firmicutes and relatively lower proportions of Bacteroidetes in DIO mice when compared with lean mice (p-value ≤0.05). At family level, significantly greater proportions of *Rhodospirillaceae*, *Lachnospiraceae*, *Streptococcaceae*, *Lactobacillaceae*, and *Clostridiaceae* in DIO mice were apparent when compared to lean mice (p value ≤0.05), whereas significantly lower proportions of *Alcaligenaceae*, *Rikenellaceae*, *Bacteroidaceae* and *Coriobacterineae* were evident when DIO mice were compared with lean mice (p value ≤0.05). At genus level, when the microbiota of DIO mice were compared with that of lean mice, significantly more, *Thalassospira*, *Alistipes*, *Odoribacter* and *Bacteroides* and significantly less *Sutterella Lactococcus*, *Turicibacter*, *Lactobacillus*, *Clostridium* and *Anaeroplasma* were observed (p value ≤0.05; Figure 3 & Table S1 in File S1).

High-throughput DNA sequence based analysis of the gut microbiota at week 2 also revealed statistically significant differences at phylum level between DIO and DIO vancomycin treated mice. Significant decreases in proportions of Bacteroidetes and Deferibacteres (p value ≤0.05), but not Firmicutes, were noted in vancomycin treated DIO mice compared with DIO mice. An increase in proportions of Proteobacteria (p value ≤0.05) in DIO vancomycin treated mice was also evident. At family level, proportions of *Rhodospirillaceae*, *Rikenellaceae*, *Porphyromonadaceae*, *Bacteroidaceae*, *Ruminococcaceae*, *Peptostrepococcaceae*, *Peptococcaceae*, *Erysipelotrichaceae*, and *Deferribacteraceae* (p value ≤0.05) were all relatively lower in the vancomycin treated mice relative to DIO controls whereas proportions of *Alcaligenaceae*, *Enterobacteriaceae, Streptococcaceae*, *Lactobacillaceae* and *Leuconostocaceae* (p value ≤0.05) were all relatively greater in the former group. At genus level relatively lower proportions of *Thalassospira*, *Alistipes, Rikenella, Parabacteroides, Odoribacter, Bacteroides, Lachnospiraceae Incertae Sedis, Coprococcus, Ruminococcaceae Incertae Sedis, Oscillibacter, Anaerotruncus, Turicibacter, Allobaculm, Mucispirillum,* uncultured *Lachnospiraceae* genus members, *Peptostreptococcaceae Incertae Sedis, Peptococcus, Clostridium and Anaeroplasma* (p value ≤0.05) were detected in DIO vancomycin treated mice compared with DIO mice. These were accompanied by the presence of relatively greater proportions of *Sutterella*, *Lactococcus*, *Lactobacillus*, *Weissella* and members of *Enterobactereaceae*-associated genera in the antibiotic treated group (p value ≤0.05) (Figure 3 & Table S1 in File S1).

The sequence data generated was investigated from a temporal perspective. As suggested from α diversity values, the six weeks which passed from intervention week 2 to 8 of the study impacted on microbiota composition even in animals where no intervention occurred, i.e. the lean and DIO controls, presumably as a consequence of the aging of the animals. At week 8, among the lean animals, it was noted that significant increases in proportions of the phyla Deferribacteres and Verrucomicrobia (p value ≤0.05) occurred and, at family level, the relative proportions of *Rhodospirillaceae, Desulfovibrionaceae, Deferribacteraceae, Lactobacillaceae, Verrucomicrobiaceae* and *Eubacteriaceae* (p value ≤0.05) were all significantly increased when compared with microbiota from the same animals at week 2. At genus level proportions of *Thalassospira, Desulfovibrio, Allobaculm, Mucispirillum, Lactobacillus, Akkermansia, Bilophila* and *Blautia* (p value ≤0.05) all increased between weeks 2 and 8. In contrast, proportions of Bacteroidetes (phylum, p value ≤0.05), *Porphyromonadaceae, Bacteroidaceae, Ruminococcaceae* (family, p value ≤0.05) and *Alistipes, Bacteroide, Coprococcus and Ruminococcaceae Incertae Sedis* (genus, p value ≤0.05) all decreased between the two time points.

Among DIO mice there were relative increases in the proportions of Proteobacteria, Actinobacteria, Deferribacteres and Verrucomicrobia (p value ≤0.05) at the week 8, relative the week 2 time point. This corresponded to significant increases in *Rhodospirillaceae, Desulfovibrionaceae, Bifidobacteriaceae, Deferribacteraceae* and *Verrucomicrobiaceae* at the family level (p value ≤0.05) and *Desulfovibrio, Mucispirillum,* uncultured *Lachnospiraceae*, *Akkermansia, Bilophila* and *Catabacter* (p value ≤0.05) at the genus level. In contrast, proportions of *Ruminococcaceae*, *Clostridiaceae* (p value ≤0.05) and *Coprococcus*, *Ruminococcaceae Incertae Sedis*, *Turicibacter* and *Clostridium* decreased between the two time points (p value ≤0.05).

Comparison of the impacts of vancomycin treatment at week 2 relative to those at week 8 had the potential to be particularly revealing given that the relative extent to which the antibiotic impacted on weight gain was greater at the earlier time point and that α diversity also increased during the intervening period. This suggested that a compensatory effect, possibly due to the recovery of specific populations, occurred between the two time points. Analysis revealed that several taxa increased in relative proportions during this interval. These included Bacteroidetes, Actinobacteria, Defferibacteres and Verrucomicrobia (p value ≤0.05) at the phylum level, *Desulfovibrionaceae, Rikenellaceae, Porphyromonadaceae, Ruminococcaceae, Erysipelotrichaceae, Bifidobacteriaceae, Deferribacteraceae, Verrucomicrobiaceae* and *Enterobacteriaceae* (p value ≤0.05) at the family level and *Ruminococcaceae Incertae Sedis, Turicibacter, Clostridium, Akkermansia, Allobaculum, Desulfovibrio, Alistipes*, *Bifidobacterium, Mucispirillum, Anaeroplasma* and members of *Enterobacteriaceae-*associated genera (p value ≤0.05) at the genus level. Of the genus level changes, only the *Ruminococcaceae Incertae Sedis, Turicibacter, Clostridium* and *Akkermansia* associated changes corresponded with temporal changes which were evident in the DIO control group and thus the other genera which underwent relative increases in proportions likely represent the populations which have most successfully adapted to vancomycin exposure. Within the vancomycin treatment group the only phylum to decrease significantly, from a temporal perspective, in the gut microbiota of the vancomycin administered mice was the Firmicutes (p value ≤0.05), which corresponded with a significant decrease in the relative proportions of the family *Streptococcaceae* (p value ≤0.05) and the genus *Lactococcus* (p value ≤0.05). A significant decrease over time was also apparent within the genus *Weissella* (p value ≤0.05) (Table S1 in File S1).

UCC118 Bac^+^ associated reductions in weight gain correspond to reductions in Rikenellaceae and Porphyromonadaceae and increases in Peptococcaceaepopulations.

**Figure 4:**
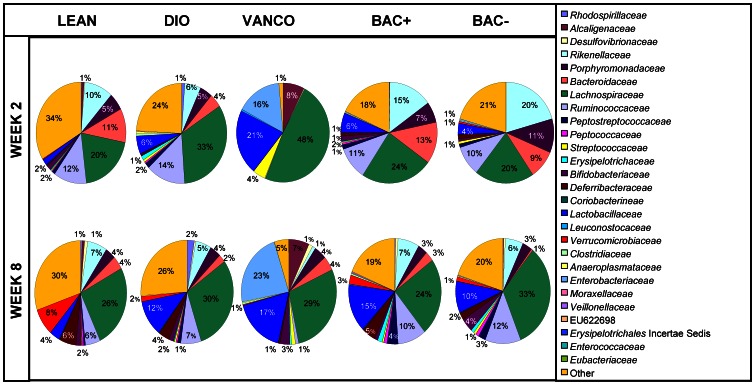
Microbial distribution at family level. Family level microbial distribution in all data sets at intervention week 2 and week 8. The pie charts represent total percentage read number for the corresponding colour coded family (*n* = 9–10 per group).

While the impact of vancomycin administration on weight gain decreased from intervention week 2 to week 8 of the study, this impact continued to be significant throughout. However, the impact on weight gain of employing the bacteriocin producing probiotic relative to its isogenic non-bacteriocin producing equivalent changed from being significant to non-significant over the same duration. Comparison of the impact of the two strains at week 2 revealed no significant differences at the phylum level between the two groups. However, at family level, a relative decrease in proportions of *Rikenellaceae* and *Porphyromonadaceae* (p value ≤0.05) was noted in Bac^+^ mice relative to their Bac^-^ counterparts. At genus level, a relative reduction was observed in *Alistipes* (p value ≤0.05) in Bac^+^ mice compared with Bac^-^ mice. Furthermore, a relative increase in proportions of the family *Peptococcaceae* (p value ≤0.05) and the corresponding genus *Peptococcus* (p value ≤0.05) was noted in Bac^+^ mice compared with Bac^-^ mice (Figure 4 & Table S2 in File S1).

Continued exposure to the bacteriocin producing UCC118 strain brought about a variety of changes. At phylum level the relative proportions of Firmicutes, Candidate Division TM7, Deferribacters and Verrucomicrobia (p value ≤0.05) were significantly increased by week 8. Proportions of the families *Desulfovibrionaceae, Erysipelotrichaceae, Deferribacteraceae, Verrucomicrobiaceae* and *Eubacteriaceae* (p value ≤0.05) and the genera *Desulfovibrio, Lactococcus, Akkermansia, Clostridium, Bilophila, Mucispirillum, Turicibacter and Catabacter* (p value ≤0.05) also increased. Over the same period relative reductions in the phylum Bacteroidetes (p value ≤0.05), the families *Rikenellaceae, Porphyromonadaceae* and *Bacteroidaceae* (p value ≤0.05) as well as the genera *Alistipes, Parabacteroides* and *Bacteroides* (p value ≤0.05) also occurred (Figure 4 & Table S2 in File S1).

The impact of administering the bacteriocin negative UCC118 strain also changed between the two time points. At phylum level relative increases in the proportions of the Firmicutes, Actinobacteria and Candidate Division TM7 (p value ≤0.05) were seen which corresponded with increases at family level in *Desulfovibrionaceae, Lachnospiraceae, Peptococcaceae, Erysipelotrichaceae, Bifidobacteriaceae, Coriobacterineae* and *Lactobacillaceae* (p value ≤0.05). At genus level increases in *Desulfovibrio, Turicibacter, Bifidobacterium, uncultured Lachnospiraceae, Peptococcus, Bilophila* and *Catabacter* (p value ≤0.05) were also noted. Relative reductions in the phylum Bacteroidetes (p value ≤0.05), families *Rikenellaceae, Porphyromonadaceae* and *Bacteroidaceae* (p value ≤0.05) as well as the genera *Alistipes, Rikenella, Parabacteroides* and *Bacteroides* (p value ≤0.05) also occurred (Figure 4 & Table S2 in File S1). It should also be noted that the proportions of *Rikenellaceae, Porphyromonadaceae and Peptococcaceae* in Bac^+^ fed mice relative to that in Bac^–^ fed controls did not differ significantly at the later time point (intervention week 8).

### Vancomycin Administration Reduces Total Bacterial Numbers in the Gut

**Figure 5:**
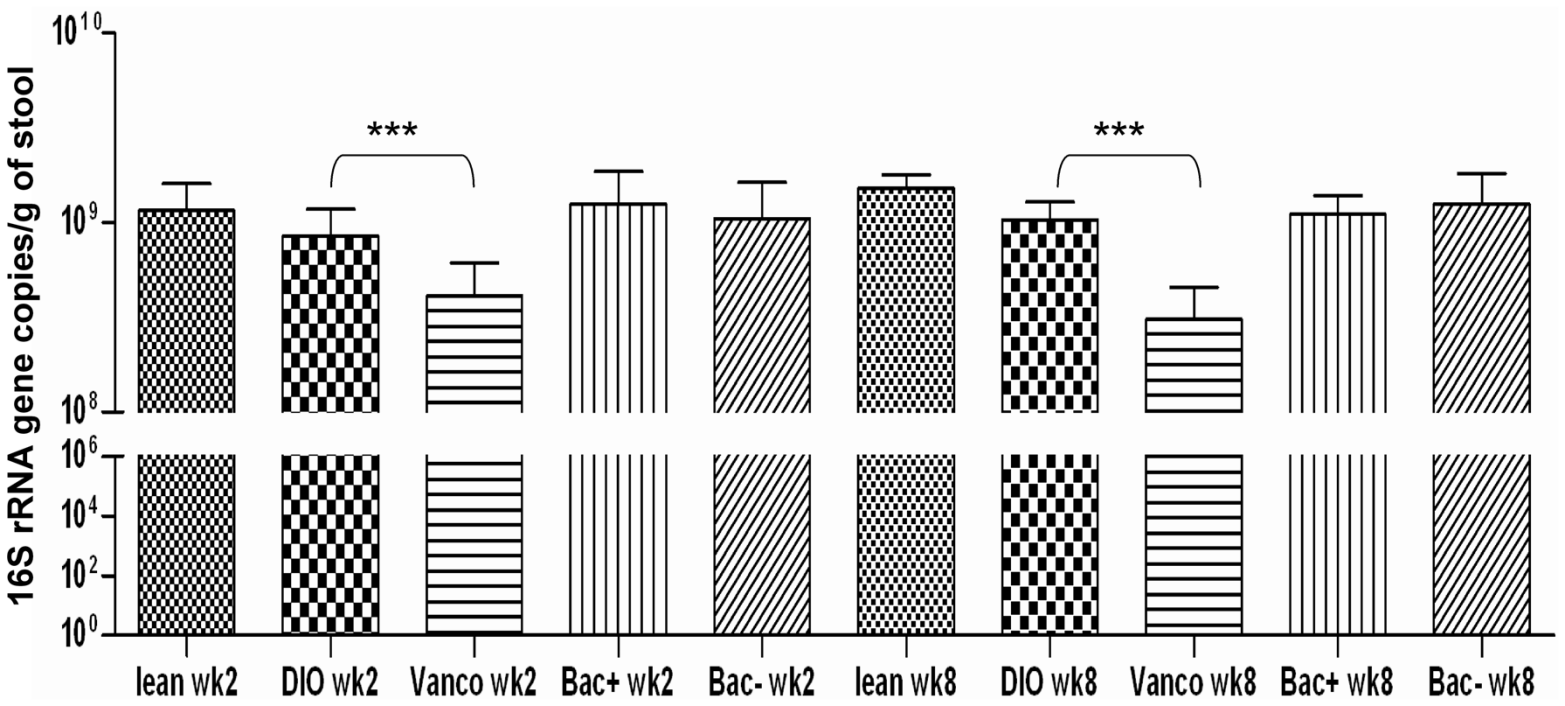
Total bacterial number observed in all treatment groups at both time points. Quantitative PCR reveals that the changes occurring are qualitative not quantitative as no significant difference is observed between time points. Total bacterial numbers calculated as copies of 16 S rRNA/g wet stool. Statistical significant difference between treatment groups is denoted by ***. p value based on kruskal wallis analysis with statistical significant determined as p≤0.05. Error bars represent the standard error of the mean.

As high throughput sequencing reveals information with respect to relative proportions of populations rather than relative numbers, quantitative PCR was employed to determine if the antimicrobial employed, i.e. vancomcyin or the bacteriocin Abp118, impacted on the total number of gut microbes present. Analysis revealed that there were no significant differences in total bacterial numbers in the faeces of diet induced obese mice compared with lean controls (p<0.96) at week 2. While treatment of the diet-induced obese mice with vancomycin resulted in a decrease in absolute faecal bacteria compared with their diet-induced obese counterparts (P<0.006 Figure 5), bacteriocin production did not alter the total bacterial numbers in the diet induced obese mice. The significant impact of vancomycin, but not other interventions, on total bacterial counts is also apparent at week 8. [7].

### Beta Diversity Highlights Temporal Variation in Microbial Populations

**Figure 6:**
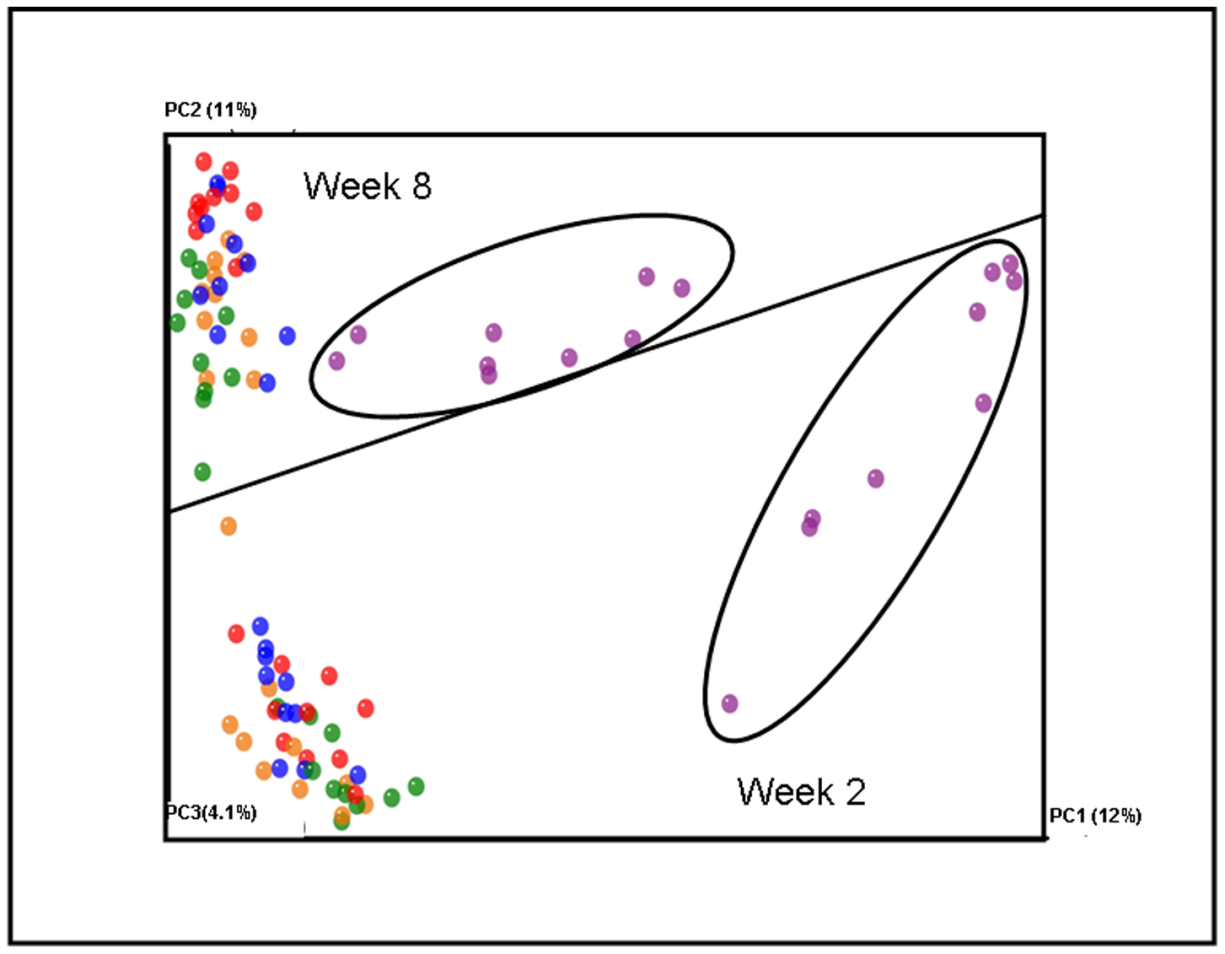
Principal coordinate analysis of unweighted unifrac reveals temporal shift. Vancomycin treated diet induced obese mice (purple) cluster away from other groups at both time points, however the distance between these mice and other treatment groups at week 8 is less than at week 2. Data sets: purple vancomycin, blue lean, red DIO, green Bac^+^ and orange Bac^-^.

Principal coordinate analysis (based on unweighted unifrac distances) of the 16 S rRNA sequences further highlights the temporal changes in the microbial populations from intervention week 2 to 8 with samples clearly clustering according to time point (Figure 6). In line with the α diversity and taxonomical data presented above, it is apparent that data points corresponding to DIO mice who received vancomycin (purple) cluster away from those corresponding to the other groups. The degree to which these data points are removed is more apparent at week 2, again suggesting that a recovery occurs during the subsequent weeks (Figure 6). At week 8, data points corresponding to the DIO controls (red) cluster tightly together within a larger cluster. Such tight clustering is not apparent at week 2 (Figure 6). It would also appear that the Bac^+^ (green) and Bac^-^ (orange) mice are more distinct at week 2 (Figure 6). Hierarchical clustering of the OTUs from each dataset also highlights the temporal instability in the mouse microbiota between the two time points. However the unweighted pair group method with arithmetric mean (UPGMA) tree shows that, at specific time points, the microbiota of mice within each treatment group are generally more similar to each other than they are to those from the other groups (Figure S3). Hierarchical clustering again highlights the separation of the vancomycin-exposed populations from the other groups. In addition to clustering away from the other groups, it is also clear that these populations differ at the respective time points. At week 8, the ‘lean’ OTUs cluster into two groups on either side of the DIO OTUs, this separation was not observed at week 2. No significant difference in weight was observed between these respective lean subgroups. At week 2 Bac^+^ OTUs are divided into two groups on either side of DIO OTUs. This separation did not persist with time.

## Discussion

This analysis of the gut microbiota of animals subjected to either vancomcyin or the bacteriocin producing probiotic *L. salivarius* UCC118 intervention provides valuable information regarding the temporal nature of the resultant changes. The results reflect microbial adaptation over time and the resilience of the microbiota. Principal coordinate analysis (PCoA) and hierarchical clustering analysis reveal that clustering occurs as a feature of time and to a lesser extent, treatment groups, rather than between microbial populations from within the same animal. In agreement with previous studies, [22], [23], [24] we established that the diet-induced obesity-associated murine gut microbiota differed from that of lean controls. More specifically, a significant increase in the proportion of Firmicutes and a decrease in the proportion of Bacteroidetes. Vancomycin was selected because of its limited systemic impact and its apparent ability to specifically target the low GC Gram-positive organisms i.e. Firmicutes. [7] Here, we establish that treatment of mice on a high fat diet with vancomycin resulted in significant alterations in the composition of the gut microbiota; including a decrease in the relative proportions of Bacteroidetes and Deferribacteres and a relative increase in the proportions of Proteobacteria relative to DIO controls. This vancomycin-induced effect on Proteobacteria populations has been noted before. [25], [26] In contrast to week 8, the proportion of Firmicutes was not reduced between animals receiving vancomycin and controls at intervention week 2. At this time point, the corresponding increased proportions of the families *Streptococcaceae, Lactobacillaceae* and *Leuconostocaceae* (all members of the Firmicutes phylum) might negate the detrimental impact of vancomycin on other Firmicutes members, resulting in the absence of an overall net change. The glycopeptide antibiotic vancomycin is traditionally known to be active against Gram-positive bacteria such as *Staphylococcus aureus* and *Clostridium difficile* through the inhibition of cell wall synthesis. [25], [27], [28] The cell wall of Gram-negative bacteria is protected by the presence of an outer membrane that blocks the effects of vancomycin. [29] Nevertheless, here we found that eight genera (*Thalassospira, Alistipes, Rikenella, Parabacteroides, Odiorbacter, Bacteroides, Oscillibacter* and *Mucispirillum*) of Gram-negative bacteria were significantly reduced in vancomycin treated DIO mice at week 2. Also three Gram-positive genus (*Lactococcus*, *Lactobacillus* and *Weissella*) increased at week 2 in vancomycin treated mice. While the innate vancomycin resistance of many lactobacilli has been well established, there have been rare reports of resistant *Lactococcus* and *Weissella* isolates. [30], [31], [32] Other differences between the gut microbial populations of these mice at week 2 and week 8 were also apparent. These included significant increases in the relative proportions of Bacteroidetes, Actinobacteria, Deferribacteres and Verrucomicrobia and a significant decrease in the relative proportions of Firmicutes at the later time points. The recovery of both Gram-positive (*Turicibacter, Ruminococcaceae Incertae Sedis, Clostridium, Allobaculum and Anaeroplasma*) and Gram-negative bacteria by week 8 (*Alistipes and Mucispirillum*) highlights the resilience of the gut bacteria. On the basis of PCoA and hierarchical clustering these temporal changes seems to represent a recovery of/development of resistance among the gut microbiota such that it less considerably differs from that of controls. While it is tempting to speculate that this reflects the emergence of resistant strains from among these populations, further investigations are required to definitively establish the basis for this recovery. This recovery coincides with vancomycin having a relatively less dramatic impact on weight gain by week 8 of the study but the identity of the population that may be contributing to this phenomenon is difficult to ascertain due to the numbers of different taxa which are altered, however it is also apparent that the total number of bacteria did not alter between the two time points. These results highlight the resilience of the microbiota to change, demonstrating that after the initial impact from vancomycin they start to revert back to their original profile. This highlights the challenge faced when utilising antimicrobials, prebiotics, [33] microbial transplantation [34] or other interventions in order to bring about long-term changes to the obesity-associated (and other) gut microbial populations. Indeed, the temporal resilience of the gut microbiota following exposure to antibiotics has been highlighted in previous studies. [14], [35], [36], [37], [38], [39].

The comparison of the impact of Bac^+^ and Bac^-^ on the murine microbiota may be more revealing as a consequence of the number of changes being fewer and the fact that the impact of Bac^+^ intervention changed from being significant to non-significant as the study continued. While previous studies with bacteriocin-producing UCC118 strain have shown it to be active against representatives of several Gram-positive taxa, including *Bacillus*, *Listeria monocytogenes*, *Enterococcus*, *Staphylococcus* and *Clostridium perfringens* [40], [41], high throughput sequencing again provided unexpected results with respect to compositional changes, at family level, relative to Bac^-^ controls at week 2. Specifically a relative reduction in the proportions of the Gram-negative families *Rikenellaceae* and *Porphyromonadaceae* and a relative increase in the proportions of the Gram-positive *Peptococcaceae* occurred. Interestingly, a recent study has suggested a link between the *Porphyromonadaceae* with the development of metabolic syndrome. [42] While the decrease in weight gain observed in Bac^+^, relative to Bac^-^, mice at week 2 and the reduction in relative numbers of this family is notable, the proportions of this family decreased even further in Bac^+^ mice by week 8 despite the fact that the impact of the probiotic with respect to weight gain was no longer significant by this time. Notably the proportions of *Rikenellaceae, Porphyromonadaceae and Peptococcaceae* in Bac^+^ and Bac^–^ fed mice did not differ significantly at week 8. Further studies are needed to explore the role these families play in the link between the gut microbial ecosystem and obesity.

In conclusion, the data demonstrate that though vancomycin distinctively modified the gut microbiota composition, the growth of some bacterial families over time may be responsible for this intervention having a less dramatic impact on weight gain by the end of the 8 week intervention period. There also exist a number of changes in the microbial population of animals administered Bac^+^ which, if successfully targeted over a longer period, could potentially extend the duration over which weight gain is significantly reduced. These results provide further rationale for altering the gut microbiota using antimicrobials but the specific identification of the populations involved and the specificity of action of the antimicrobials will be essential and may require continual modification to ensure a sustained impact and to overcome compensatory effects.

## Supporting Information

Figure S1 **Experimental design.** Seven week old C57BL/J6 mice were fed a high fat or low fat diet for 20 weeks, after 12 weeks intervention began. Sequencing was performed at intervention week 2 and week 8 of the study.
(TIF)

Figure S2 **Rarefaction curves for each group at 97% similarity levels for intervention week 2 (A) and week 8 (B) data sets.** Amount of operational taxonomic units (OUT’s) found as a function of the number of sequence tags sampled.
(TIF)

Figure S3 **Unweighted pair group method with arithmetic mean (UPGMA) tree of all samples at both time point’s.** Highlights temporal shift and clustering by treatment group. Vancomycin treated DIO mice present as outliers from both time points.
(TIF)

File S1 **Contains Table S1 and Table S2. Table S1.** Vancomycin treatment alters gut microbiota in diet induced obese mice. **Table S2**. Effects of *L salivarius* UCC118 bacteriocin production on the gut microbiota of DIO mice over time.
(DOC)
