# Supplementary figures and images for: Targeting the Microbiota to Address Diet-Induced Obesity: A Time Dependent Challenge
Source: PLoS One. 2013 Jun 7;8(6):e65790. doi: 10.1371/journal.pone.0065790 (PMC3676335; doi:10.1371/journal.pone.0065790)

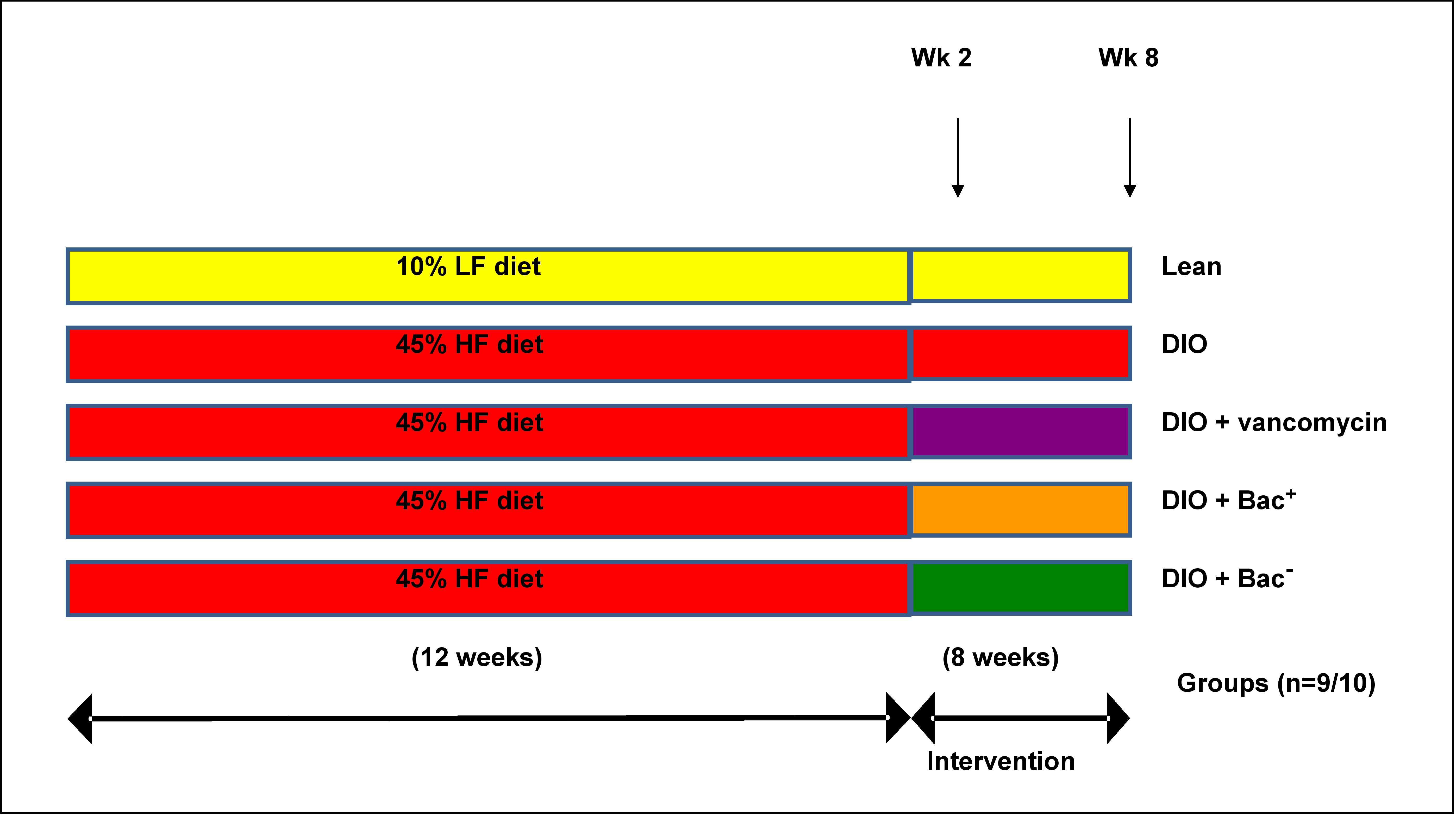

Supplement: Figure S1 — Experimental design. Seven week old C57BL/J6 mice were fed a high fat or low fat diet for 20 weeks, after 12 weeks intervention began. Sequencing was performed at intervention week 2 and week 8 of the study. (TIF) [file pone.0065790.s001.tif]

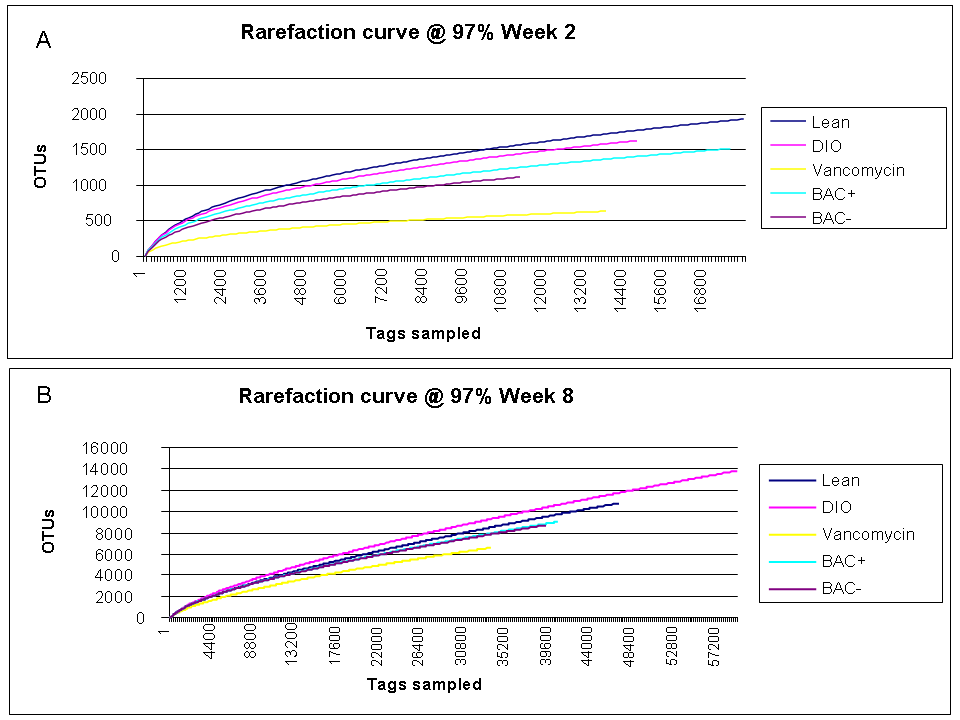

Supplement: Figure S2 — Rarefaction curves for each group at 97% similarity levels for intervention week 2 (A) and week 8 (B) data sets. Amount of operational taxonomic units (OUT’s) found as a function of the number of sequence tags sampled. (TIF) [file pone.0065790.s002.tif]

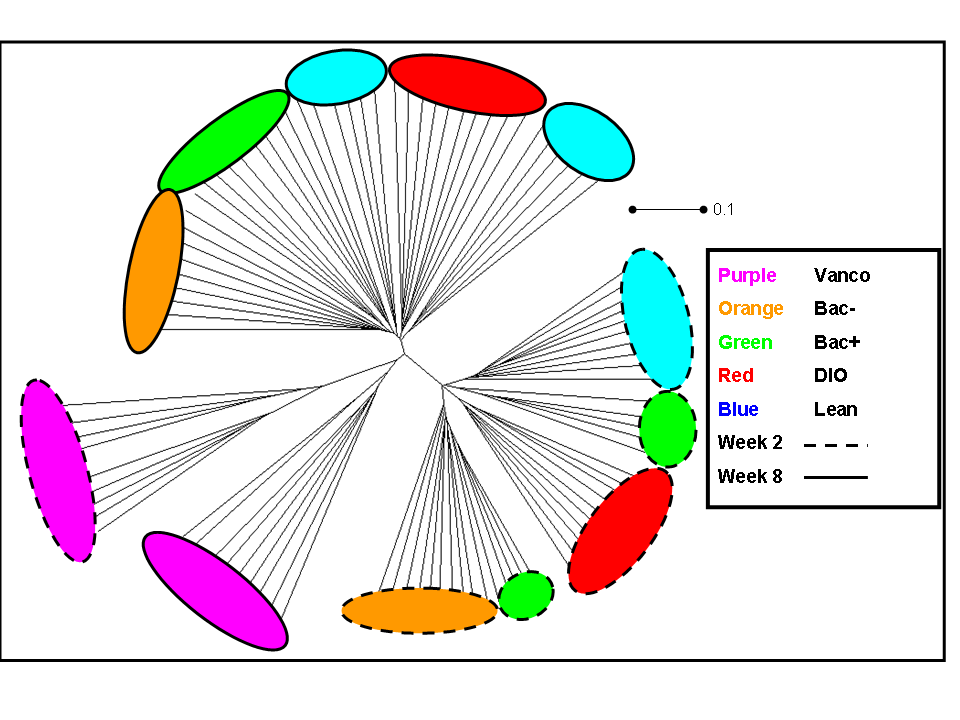

Supplement: Figure S3 — Unweighted pair group method with arithmetic mean (UPGMA) tree of all samples at both time point’s. Highlights temporal shift and clustering by treatment group. Vancomycin treated DIO mice present as outliers from both time points. (TIF) [file pone.0065790.s003.tif]
