# Supplementary material for: Targeting the Microbiota to Address Diet-Induced Obesity: A Time Dependent Challenge
Source: PLoS One. 2013 Jun 7;8(6):e65790. doi: 10.1371/journal.pone.0065790 (PMC3676335; doi:10.1371/journal.pone.0065790)
Supplement: File S1 — Contains Table S1 and Table S2. Table S1. Vancomycin treatment alters gut microbiota in diet induced obese mice. Table S2. Effects of L salivarius UCC118 bacteriocin production on the gut microbiota of DIO mice over time. (DOC) [file pone.0065790.s004.doc]

**Table S1.** Vancomycin treatment alters gut microbiota in diet induced obese mice

|  | **Lean**  **Wk 2** | **Lean**  **Wk 8** | **DIO**  **Wk 2** | **DIO**  **Wk 8** | **Vancomycin Wk 2** | **Vancomycin Wk 8** |
| --- | --- | --- | --- | --- | --- | --- |
| **Phylum** |  |  |  |  |  |  |
| Proteobacteria | 1.51± 0.22 | 2.43±0.37 | 1.28±0.28† | 3.18±0.83 | 24.63±6.90* | 31.74±1.90 |
| Bacteroidetes | 42.89± 2.48# | 21.50±2.13## | 22.13±4.69 | 16.82±1.26 | 0.67±0.45* | 2.24±0.76** |
| Firmicutes | 52.92± 2.65# | 60.03±2.90 | 74.96±4.54 | 72.06±2.05 | 74.18±7.15 | 61.49±2.61** |
| Actinobacteria | 1.54± 0.66 | 2.20±0.61 | 0.58±0.22† | 1.86±0.46 | 0.34±0.31 | 3.06±1.39** |
| Candidate Devision TM7 | 0.22± 0.09 | 0.27±0.05 | 0.09±0.06 | 0.15±0.08 | 0.0±0.0 | 0.01±0.01 |
| Deferribacteres | 0.53± 0.25 | 5.42±1.62## | 0.61±0.18† | 3.86±1.28 | 0.0±0.0* | 0.60±0.27** |
| Verrucomicrobia | 0.10±0.10 | 7.90±3.11## | 0.0±0.0† | 1.65±0.45 | 0.0±0.0 | 0.20±0.08** |
| 4COd 2 | 0.0±0.0 | 0.0±0.0 | 0.07±0.07 | 0.14±0.05 | 0.0±0.0 | 0.0±0.0 |
| **Family** |  |  |  |  |  |  |
| *Rhodospirillaceae* | 0.16±0.06# | 0.54±.08## | 0.87±0.28**†** | 2.38±0.77 | 0.0±0.0* | 0.01±0.01 |
| *Alcaligenaceae* | 0.94± 0.22# | 0.99±0.42 | 0.0±0.0 | 0.03±0.02 | 7.74±3.09* | 6.87±0.91 |
| *Desulfovibrionaceae* | 0.24±0.11 | 0.82±0.22## | 0.09±0.07**†** | 0.71±0.14 | 0.07±0.05 | 1.42±0.29** |
| *Rikenellaceae* | 10.55±1.01# | 7.61±1.18 | 5.93±1.46 | 5.38±0.79 | 0.20±0.20* | 1.10±0.36** |
| *Porphyromonadaceae* | 5.50±0.59 | 3.72±0.42## | 4.71±1.44 | 3.92±0.54 | 0.0±0.0* | 3.81±3.68** |
| *Bacteroidaceae* | 10.40±1.65# | 4.38±0.45## | 4.46±2.08 | 2.45±0.45 | 0.17±0.17* | 5.85±5.74 |
| *Lachnospiraceae* | 20.83±2.03# | 24.97±2.61 | 32.50±4.25 | 29.87±2.09 | 48.27±11.82 | 27.52±5.98 |
| *Ruminococcaceae* | 12.13±0.97 | 5.62±0.99## | 13.58±1.46**†** | 7.02±1.17 | 0.20±0.13* | 1.14±0.45** |
| *Peptostreptococcaceae* | 0.96±0.23 | 0.51±0.24 | 1.67±05.6 | 1.19±0.41 | 0.0±0.0* | 0.04±0.03 |
| *Peptococcaceae* | 0.39±0.09 | 0.36±0.05 | 0.56±0.15 | 0.51±0.11 | 0.0±0.0* | 0.06±0.03 |
| *Streptococcaceae* | 0.37±0.09# | 0.34±0.05 | 0.78±0.16 | 0.58±0.07 | 4.88±1.69* | 1.34±0.14** |
| *Erysipelotrichaceae* | 0.09±0.06 | 0.16±0.08 | 1.43±0.74 | 0.11±0.06 | 0.0±0.0* | 0.63±0.29** |
| *Bifidobacteriaceae* | 1.33±0.61 | 2.03±0.60 | 0.56±0.22**†** | 1.67±0.40 | 0.34±0.31 | 3.45±1.34** |
| *Deferribacteraceae* | 0.53±0.25 | 5.42±1.62## | 0.48±0.17**†** | 3.86±1.28 | 0.0±0.0* | 0.61±0.27** |
| *Coriobacterineae* | 0.13±0.07# | 0.14±0.04 | 0.0±0.0**†** | 0.18±0.06 | 0.0±0.0 | 0.0±0.0 |
| *Lactobacillaceae* | 1.970±0.51# | 3.88±0.69## | 6.27±1.67 | 11.82±2.43 | 19.27±6.21* | 16.52±3.54 |
| *Leuconostocaceae* | 0.05±0.05 | 0.04±0.02 | 0.0±0.0 | 0.04±0.02 | 0.40±0.13* | 0.08±0.03 |
| *Verrucomicrobiaceae* | 0.10±0.10 | 7.90±3.11## | 0.0±0.0**†** | 1.65±0.45 | 0.0±0.0 | 0.20±0.08** |
| *Clostridiaceae* | 0.0±0.0# | 0.01±0.01 | 0.82±0.33**†** | 0.0±0.0 | 0.13±0.09 | 0.73±0.31 |
| *Enterobacteriaceae* | 0.0±0.0 | 0.02±0.02 | 0.0±0.0 | 0.01±0.01 | 16.64±3.87* | 23.32±1.20** |
| *EU622698* | 0.0±0.0 | 0.0±0.0 | 0.06±0.06 | 0.09±0.03 | 0.0±0.0 | 0.0±0.0 |
| *Eubacteriaceae* | 0.0±0.0 | 0.06±0.03## | 0.0±0.0 | 0.02±0.01 | 0.0±0.0 | 0.0±0.0 |
| **Genus** |  |  |  |  |  |  |
| *Thalassospira* | 0.16±0.06# | 0.47±0.07## | 0.87±0.28 | 1.59±0.33 | 0.0±0.0* | 0.01±0.01 |
| *Sutterella* | 0.94±0.22# | 0.99±0.42 | 0.0±0.0 | 0.03±0.02 | 7.74±3.09* | 6.87±0.91 |
| *Desulfovibrio* | 0.20±0.10 | 0.65±0.21## | 0.04±0.04† | 0.50±0.11 | 0.07±0.05 | 1.41±0.28** |
| *Alistipes* | 7.53±0.88# | 4.01±0.42## | 3.52±0.98 | 2.43±0.25 | 0.13±0.13* | 0.77±0.26** |
| *Rikenella* | 2.06±0.32 | 2.82±0.76 | 1.71±0.67 | 1.89±0.63 | 0.0±0.0* | 0.0±0.0 |
| *Parabacteroides* | 1.82±0.47 | 1.67±0.12 | 3.45±1.14 | 2.75±0.50 | 0.0±0.0* | 0.03±0.02 |
| *Odoribacter* | 3.68±0.66# | 2.05±0.39 | 1.24±0.34 | 1.16±0.16 | 0.0±0.0* | 0.04±0.03 |
| *Bacteroides* | 10.40±1.65# | 4.38±0.45## | 4.46±2.08 | 2.45±0.45 | 0.17±0.17* | 0.13±0.07 |
| *Lachnospiraceae Incertae Sedis* | 0.66±0.13 | 0.41±0.07 | 0.70±0.20 | 0.59±0.10 | 0.0±0.0* | 0.10±0.06 |
| *Coprococcus* | 0.40±0.11 | 0.10±0.04## | 0.58±0.14† | 0.17±0.04 | 0.0±0.0* | 0.02±0.02 |
| *Ruminococcaceae Incertae Sedis* | 6.66±0.62 | 2.12±0.36## | 7.16±0.74† | 3.09±0.43 | 0.0±0.0* | 0.56±0.22** |
| *Oscillibacter* | 0.54±0.14 | 0.73±0.19 | 0.84±0.16 | 0.99±0.16 | 0.0±0.0* | 0.12±0.07 |
| *Anaerotruncus* | 1.54±0.24 | 1.06±0.20 | 2.27±0.37 | 1.47±0.24 | 0.0±0.0* | 0.12±0.06 |
| *Lactococcus* | 0.35±0.08# | 0.33±0.05 | 0.74±0.15 | 0.56±0.07 | 4.76±1.67* | 1.33±0.14** |
| *Turicibacter* | 0.09±0.06# | 0.16±0.08 | 1.73±0.70† | 0.11±0.06 | 0.0±0.0* | 0.63±0.29** |
| *Allobaculum* | 12.77±1.61 | 21.18±3.34## | 10.94±2.80 | 17.09±2.94 | 0.13±0.13* | 2.63±1.00** |
| *Bifidobacterium* | 1.36±0.61 | 2.03±0.60 | 0.56±0.22 | 1.46±0.42 | 0.34±0.31 | 3.45±1.34** |
| *Mucispirillum* | 0.53±0.25 | 5.42±1.62## | 0.61±0.18† | 3.86±1.28 | 0.0±0.0* | 0.61±0.27** |
| *Lactobacillus* | 1.97±0.51# | 3.85±0.69## | 6.26±1.67 | 11.74±2.41 | 19.27±6.21* | 16.49±3.53 |
| *uncultured Lachnospiraceae* | 0.19±0.08 | 0.57±0.22 | 0.33±0.17† | 2.57±0.81 | 0.0±0.0* | 0.22±0.10** |
| *Weissella* | 0.03±0.03 | 0.0±0.0 | 0.0±0.0 | 0.02±0.01 | 0.29±0.1* | 0.02±0.02** |
| *Leuconostoc* | 0.02±0.02 | 0.0±0.0 | 0.0±0.0 | 0.0±0.0 | 0.04±0.04 | 0.03±0.02 |
| *Peptostreptococcaceae Incertae Sedis* | 0.11±0.06 | 0.20±0.16 | 0.52±0.22 | 0.33±0.11 | 0.0±0.0* | 0.04±0.03 |
| *Peptococcus* | 0.09±0.10 | 0.0±0.0 | 0.34±0.15 | 0.21±0.10 | 0.0±0.0* | 0.0±0.0 |
| *Akkermansia* | 0.10±0.10 | 7.90±3.11## | 0.0±0.0† | 1.65±0.45 | 0.0±0.0 | 0.20±0.08** |
| *Clostridium* | 0.0±0.0# | 0.01±0.01 | 0.82±0.33† | 0.0±0.0 | 0.08±0.08* | 0.73±0.13** |
| *Anaeroplasma* | 0.0±0.0# | 0.02±0.02 | 0.65±0.31 | 0.06±0.03 | 0.0±0.0* | 0.07±0.04 |
| *Bilophila* | 0.0±0.0 | 0.13±0.05## | 0.03±0.03† | 0.21±0.04 | 0.0±0.0 | 0.0±0.0 |
| *Enterobacteriaceae genus* | 0.0±0.0 | 0.02±0.02 | 0.0±0.0 | 0.01±0.01 | 16.58±3.83* | 23.31±1.20** |
| *Veillonella* | 0.0±0.0 | 0.0±0.0 | 0.0±0.0 | 0.0±0.0 | 0.02±0.02 | 0.0±0.0 |
| *Streptococcus* | 0.0±0.0 | 0.0±0.0 | 0.0±0.0 | 0.0±0.0 | 0.0±0.0 | 0.0±0.0 |
| *Catabacter* | 0.0±0.0 | 0.04±0.02 | 0.0±0.0† | 0.05±0.02 | 0.0±0.0 | 0.0±0.0 |
| *Anaerovorax* | 0.0±0.0 | 0.0±0.0 | 0.0±0.0 | 0.0±0.0 | 0.0±0.0 | 0.0±0.0 |
| *Acinetobacter* | 0.0±0.0 | 0.0±0.0 | 0.0±0.0 | 0.0±0.0 | 0.0±0.0 | 0.0±0.0 |
| *Blautia* | 0.0±0.0 | 0.05±0.02## | 0.0±0.0 | 0.05±0.02 | 0.0±0.0 | 0.0±0.0 |
| *Eubacterium* | 0.0±0.0 | 0.0±0.0 | 0.0±0.0 | 0.0±0.0 | 0.0±0.0 | 0.0±0.0 |
| *Enterococcus* | 0.0±0.0 | 0.0±0.0 | 0.0±0.0 | 0.01±0.01 | 0.0±0.0 | 0.0±0.0 |

Statistical significance was determined using Kruskal Wallis. Values are mean percentage read number ± standard error. *Pvalue ≤0.05 between DIO and DIO vancomycin mice at week 2; **Pvalue ≤0.05 between DIO vancomycin mice at week 2 and DIO vancomycin mice at week 8; #Pvalue ≤0.05 between Lean and DIO mice at week 2; ##Pvalue ≤0.05 between Lean week 2 and Lean week 8; †Pvalue ≤0.05 between DIO week 2 mice and DIO week 8 mice.

**Table S2. Effects of *L salivarius* UCC118 bacteriocin production on the gut microbiota of DIO mice over time.**

|  | **BAC-**  **wk 2** | **BAC-**  **wk 8** | **BAC+**  **wk 2** | **BAC+**  **wk 8** |
| --- | --- | --- | --- | --- |
| **Phylum** |  |  |  |  |
| Proteobacteria | 0.94±0.23 | 1.00±0.12 | 0.93± 0.30 | 2.43±0.37 |
| Bacteroidetes | 48.66±4.0 | 13.4±1.6**‡** | 41.70± 1.67**†** | 21.50±2.13 |
| Firmicutes | 47.17±3.82 | 70.65±7.29**‡** | 53.84± 1.61**†** | 60.03±2.90 |
| Actinobacteria | 0.97±0.30 | 4.20±1.27**‡** | 1.85± 0.54 | 2.20±0.61 |
| Candidate Devision TM7 | 0.0±0.0 | 0.43±0.10**‡** | 0.0±0.0**†** | 0.27±0.05 |
| Deferribacteres | 1.23±0.56 | 2.40±0.72 | 1.25± 0.31**†** | 5.42±1.62 |
| Verrucomicrobia | 0.49±0.29 | 1.17±1.02 | 0.16± 0.14**†** | 7.90±3.11 |
| 4COd2 | 0.0±0.0 | 0.02±0.02 | 0.01±0.01**†** | 0.0±0.0 |
| **Family** |  |  |  |  |
| *Desulfovibrionaceae* | 0.10±0.07 | 0.58±0.10**‡** | 0.07±0.05**†** | 0.82±0.22 |
| *Rikenellaceae* | 20.99±1.88* | 6.31±0.85**‡** | 14.62±0.78**†** | 7.61±1.18 |
| *Porphyromonadaceae* | 11.15±1.43* | 3.14±0.57**‡** | 6.83±0.87**†** | 3.72±0.42 |
| *Bacteroidaceae* | 9.42±1.60 | 0.68±0.18**‡** | 12.23±1.50**†** | 4.38±0.45 |
| *Lachnospiraceae* | 19.65±2.69 | 33.38±3.55**‡** | 24.54±2.20 | 24.97±2.61 |
| *Peptococcaceae* | 0.09±0.09* | 1.21±0.21**‡** | 0.69±0.14 | 0.36±0.05 |
| *Streptococcaceae* | 0.62±0.39 | 0.60±0.11 | 0.33±0.14**†** | 0.34±0.05 |
| *Erysipelotrichaceae* | 0.0±0.0 | 1.24±0.46**‡** | 0.09±0.06**†** | 0.16±0.08 |
| *Bifidobacteriaceae* | 0.95±0.30 | 4.04±1.22**‡** | 1.78±0.52 | 2.03±0.60 |
| *Deferribacteraceae* | 1.23±0.56 | 2.46±0.70 | 1.25±0.31**†** | 5.42±1.62 |
| *Coriobacterineae* | 0.0±0.0 | 0.13±0.06**‡** | 0.02±0.02 | 0.14±0.04 |
| *Lactobacillaceae* | 3.73±0.95 | 9.71±2.16**‡** | 6.27±1.88 | 3.88±0.69 |
| *Leuconostocaceae* | 0.0±0.0 | 0.05±0.03 | 0.0±0.0**†** | 0.04±0.02 |
| *Verrucomicrobiaceae* | 0.49±0.29 | 1.17±1.02 | 0.16±0.14**†** | 7.90±3.11 |
| *Clostridiaceae* | 0.28±0.19 | 0.04±0.03 | 0.02±0.02**†** | 0.01±0.01 |
| *Anaeroplasmataceae* | 0.17±0.12 | 0.29±0.22 | 0.03±0.03 | 0.02±0.02 |
| *Enterobacteriaceae* | 0.43±0.19 | 0.16±0.03 | 0.45±0.18 | 0.02±0.02 |
| *Eubacteriaceae* | 0.0±0.0 | 0.01±0.01 | 0.0±0.0**†** | 0.06±0.03 |
| **Genus** |  |  |  |  |
| *Desulfovibrio* | 0.05±0.05 | 0.25±0.09**‡** | 0.02±0.02**†** | 0.56±0.11 |
| *Alistipes* | 13.92±1.18* | 2.97±0.53**‡** | 8.35±0.66**†** | 3.60±0.39 |
| *Rikenella* | 3.18±0.42 | 2.00±0.43**‡** | 2.64±0.48 | 2.55±0.67 |
| *Parabacteroides* | 6.80±1.11 | 1.22±.034**‡** | 3.74±0.75**†** | 1.38±0.19 |
| *Bacteroides* | 9.42±1.60 | 0.68±0.18**‡** | 12.23±1.50**†** | 2.87±0.54 |
| *Lactococcus* | 0.28±0.10 | 0.68±0.18 | 0.17±0.12**†** | 0.62±0.09 |
| *Turicibacter* | 0.0±0.0 | 1.24±0.46**‡** | 0.09±0.06**†** | 1.68±0.70 |
| *Bifidobacterium* | 0.95±0.30 | 4.04±1.22**‡** | 1.78±0.25 | 1.17±0.38 |
| *Mucispirillum* | 1.23±0.56 | 1.82±0.49 | 1.25±0.31**†** | 4.91±1.73 |
| *uncultured Lachnospiraceae* | 0.32±0.16 | 1.46±0.55**‡** | 0.54±0.19 | 1.36±0.49 |
| *Peptococcus* | 0.08±0.08* | 0.76±0.19**‡** | 0.47±0.13 | 0.51±0.14 |
| *Akkermansia* | 0.49±0.29 | 1.17±1.02 | 0.16±0.14**†** | 2.85±1.05 |
| *Clostridium* | 0.23±0.15 | 0.05±0.03 | 0.02±0.02**†** | 0.82±0.35 |
| *Bilophila* | 0.0±0.0 | 0.22±0.06**‡** | 0.01±0.01**†** | 0.36±0.11 |
| *Enterobacteriaceae genus* | 0.28±0.16 | 0.15±0.03 | 0.30±0.17 | 0.29±0.05 |
| *Catabacter* | 0.0±0.0 | 0.08±0.03**‡** | 0.0±0.0**†** | 0.11±0.04 |
| *Uncultured Bacteria* | 0.0±0.0 | 0.07±0.03**‡** | 0.01±0.01**†** | 0.21±0.05 |

Statistical significance was determined using Kruskal Wallis. Values are mean percentage read number ± standard error . *Pvalue ≤0.05 between BAC+ and BAC-at week 2; † Pvalue ≤0.05 between BAC+ week 2 and BAC+ week 8; ‡Pvalue ≤0.05 between BAC- week 2 and BAC- week 8.
